# Extracellular vesicle-functionalized bioactive scaffolds for bone regeneration

**DOI:** 10.1016/j.ajps.2024.100945

**Published:** 2024-07-11

**Authors:** Taozhao Yu, Irene Shuping Zhao, Hongguang Pan, Jianhua Yang, Huanan Wang, Yongqiang Deng, Yang Zhang

**Affiliations:** a School of Dentistry, Shenzhen University Medical School, Shenzhen 518015, China; b School of Biomedical Engineering, Shenzhen University Medical School, Shenzhen 518015, China; c Department of Otolaryngology, Shenzhen Children Hospital, Shenzhen 518034, China; d Longgang District People's Hospital of Shenzhen & The Second Affiliated Hospital, The Chinese University of Hong Kong, Shenzhen 518172, China; e Key State Laboratory of Fine Chemicals, School of Bioengineering, Dalian University of Technology, Dalian 116024, China; f Department of Stomatology, Shenzhen University General Hospital, Shenzhen University, Shenzhen 518055, China; g Institute of Stomatological Research, Shenzhen University, Shenzhen 518055, China

**Keywords:** Extracellular vesicles, Exosomes, Biomaterials, Local delivery, Bone regeneration

## Abstract

The clinical need for effective bone regeneration in compromised conditions continues to drive demand for innovative solutions. Among emerging strategies, extracellular vesicles (EVs) have shown promise as an acellular approach for bone regeneration. However, their efficacy is hindered by rapid sequestration and clearance when administered via bolus injection. To address this challenge, EV-functionalized scaffolds have recently been proposed as an alternative delivery strategy to enhance EV retention and subsequent healing efficacy. This review aims to consolidate recent advancements in the development of EV-functionalized scaffolds for augmenting bone regeneration. It explores various sources of EVs and different strategies for integrating them into biomaterials. Furthermore, the mechanisms underlying their therapeutic effects in bone regeneration are elucidated. Current limitations in clinical translation and perspectives on the design of more efficient EVs for improved therapeutic efficacy are also presented. Overall, this review can provide inspiration for the development of novel EV-assisted grafts with superior bone regeneration potential.

## Graphical abstract

**Figure:**
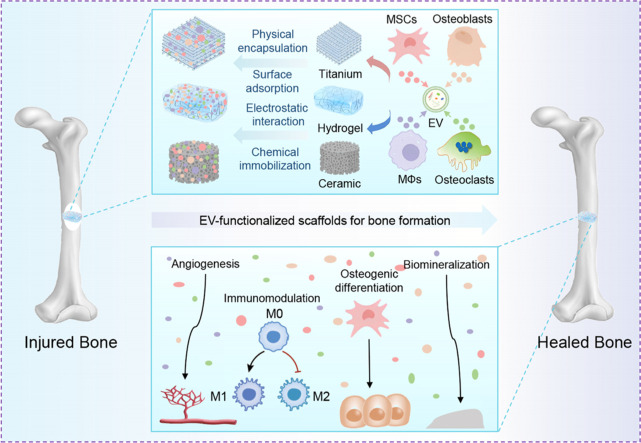

## 1 Introduction

The treatment of large bone defects caused by tumors, trauma, and infection remains a challenge in orthopedic clinics [1]. To facilitate bone regeneration, numerous strategies have been employed, including biomaterials, gene therapy and tissue engineering [2]. However, successful clinical application of these strategies still faces many limitations due to the complexity of bone regeneration process. This process involves a meticulously orchestrated sequence of events, beginning with an inflammatory response triggered by injury to attract immune cells for debris clearance, followed by mesenchymal stem cells (MSCs) differentiation into osteoblasts, promoting new bone matrix formation and angiogenesis [3]. Subsequently, the reparative phase ensues, wherein a soft callus forms at the fracture site, gradually solidifying over 2–6 weeks to provide stability. Lastly, during the remodeling stage, a fibrocartilaginous callus stabilizes the fracture site, with osteoclasts resorbing excess material and osteoblasts generating new bone tissue [4]. Throughout the bone regeneration process, intricate intercellular communication occurs between various cell types including MSCs, osteoblasts, osteoclasts, osteocytes, and immune cells. Understanding the mechanisms of action between these different cell types is critical to facilitating bone regeneration. Previous research has primarily focused on cell signaling pathways related to cytokines and cell-cell interactions [5]. In recent years, emerging research underscores the critical role of extracellular vesicles (EVs) as a key signaling pathway in regulating bone homeostasis across different stages [6].

EVs encompass a diverse array of membranous structures generated and released by various cell types. They play a crucial role in intercellular communication by transporting bioactive molecules, including microRNA (miRNA), proteins, and lipids, to specific target cells, thereby triggering specific signaling pathways. Despite their importance, categorizing EV subtypes such as exosomes, microvesicles, microparticles, endosomes, ectosomes, and multivesicular bodies can be intricate due to overlapping size ranges, marker expressions, and cargo compositions [7]. Consequently, in accordance with the International Society of Extracellular Vesicles recommendations [8], this review adopts the umbrella term "EVs" to encompass all cell-released vesicles composed of a lipid bilayer without functional nuclei.

Apart from the crucial role of EVs in bone homeostasis, the distinct advantages of low immunogenicity, sustainable sourcing, and convenient storage have facilitated their successful application in augmenting calvarial, femur, and systemic bone regeneration, offering promising alternatives to cell-based therapies. In these preclinical studies, EVs were administered through localized injection or systemic routes such as intravenous, intraperitoneal, or subcutaneous injections [9]. However, these methods lead to rapid clearance of EVs from the treatment sites and a failure to maintain an optimal concentration for therapeutic efficacy [10,11]. To address this concern, the incorporation of EVs into bioactive scaffolds becomes a potent approach to maximize their effects by increasing EV retention at the target site for extended periods [12]. Several conventional methods have been explored for incorporating EVs into biomaterials, including physical encapsulation [13], surface adsorption onto scaffolds [14], electrostatic interaction [15] or immobilization onto scaffolds by specific linker molecules [16], antibodies [17] and Extracellular matrix (ECM) components [18]. These approaches facilitate controlled release of EVs in a desired manner, enhancing their therapeutic potential.

After providing an overview of the roles of EVs in bone remodeling, this review consolidates the current landscape of EV-functionalized biomaterials utilized for bone defect repair. It delves into the various methods employed to load EVs onto biomaterials and elucidates the mechanisms through which EV-functionalized bioactive scaffolds facilitate bone regeneration. Furthermore, the review examines potential challenges and obstacles associated with EV-functionalized bioactive scaffolds in the treatment of bone diseases while proposing effective strategies to enhance bone regenerative efficacy in the future.

## 2 The significant roles of EVs in bone regeneration

**Fig. 1:**
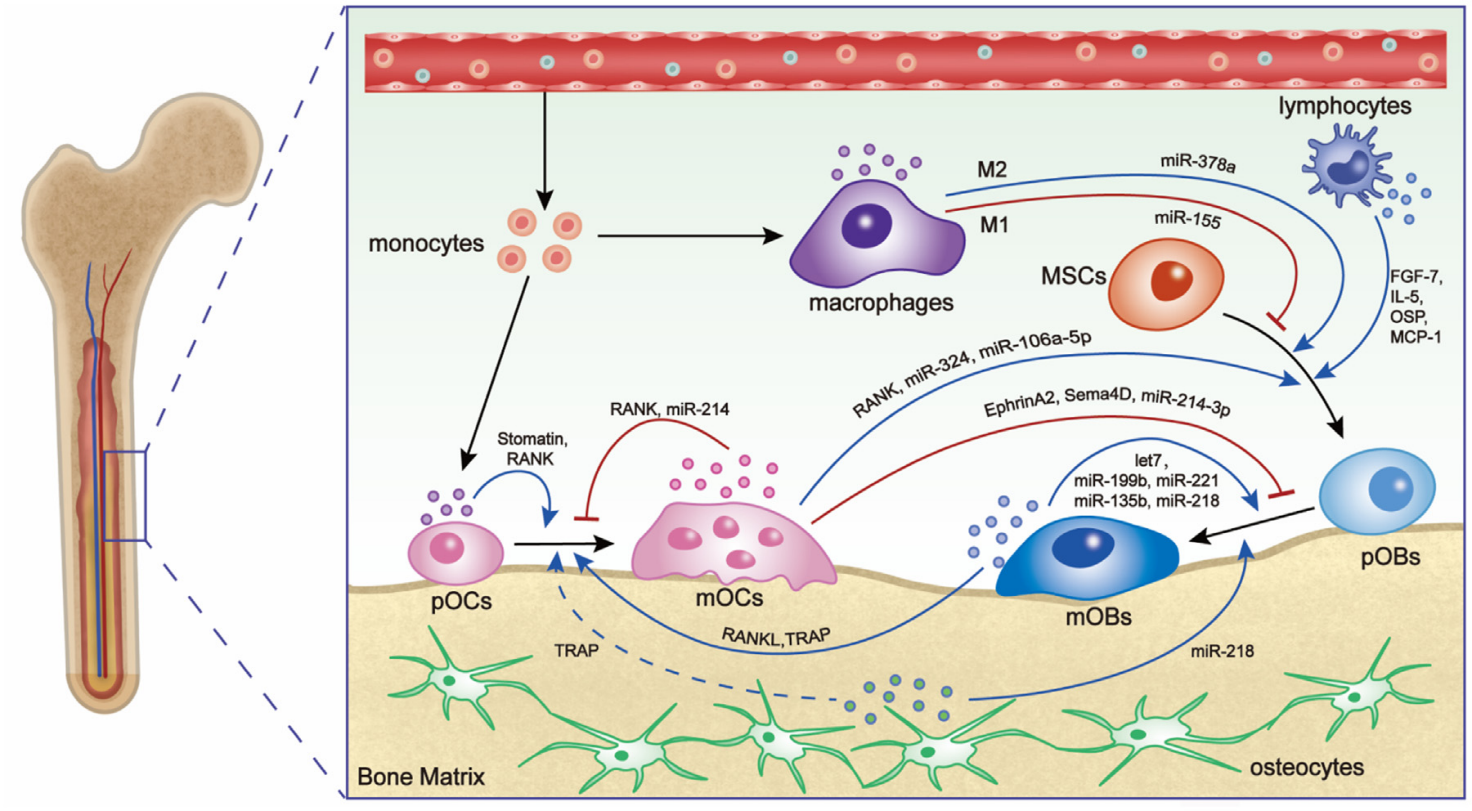
Schematic illustration of bone-related EVs in cellular communication during bone remodeling. The diagram illustrates the involvement of various miRNAs and proteins in EVs derived from MSCs, pre-osteoblasts (pOBs), mature osteoblasts (mOBs), osteoblasts, pre-osteoclasts (pOCs), mature osteoclasts (mOCs), osteocytes, monocytes, macrophages in osteogenic activity. These EVs play a crucial role in promoting (indicated by black arrows), inhibiting (indicated by red lines), and directing differentiation (indicated by black dashed arrow) pathways in response to specific EVs. The application of these EVs can be further explored to regulate the process of bone regeneration.

Bone regeneration involves a meticulously orchestrated sequence of events, with intricate intercellular communication between various cell types, including MSCs, osteoblasts, osteoclasts, osteocytes, and immune cells [19]. EVs derived from these bone-related cells and immune cells play important roles in the cellular communication involved in bone remodeling (Fig. 1). Furuta et al. [20] first demonstrated that CD9-knockout mice exhibited impaired healing of femur fractures due to reduced exosome secretion, highlighting the importance of EVs in bone regeneration. Specifically, MSC-derived EVs have been demonstrated to act as important posttranscriptional regulators of osteoblasts. In turn, osteoblasts can send messages to MSCs via EVs to establish positive feedback for bone repair and regeneration [19,21]. Moreover, the crosstalk between bone-forming osteoblasts and bone-resorbing osteoclasts also occurs through EV-mediated communication. For example, osteoblast EVs can bind to osteoclasts via RANKL-RANK signaling to facilitate osteoclast formation [22]. Conversely, osteoclasts release EVs that can affect MSCs and osteoblasts, either promoting or inhibiting osteogenesis through different cargo and signaling pathways [23,24]. Osteocytes, as other important mechanosensitive cells, play crucial roles in coordinating mechanical loading and bone formation via paracrine factors [25]. Recent findings suggested that EVs from mechanically stimulated osteocytes own significantly enhanced pro-osteogenic potentials [25]. Alongside bone-related cells, the interaction between immune cells and bone cells also determines the bone remodeling process [19,21]. EVs are released by different immune cells, such as monocytes, macrophages, and dendritic cells and can be internalized by BMSCs to promote their recruitment and differentiation [[26], [27], [28]]. In turn, EVs derived from MSCs affect the behavior of immune cells, such as acting as an immunomodulatory mediator in cellular communication [29]. For example, MSCs-derived EVs containing specific miRNAs, such as miR-451a [30], miR-1260b [31], miR-1246 [32], and miR-142-3p [33], have been found to induce anti-inflammatory macrophages or regulatory T cells to benefit bone repair.

## 3 Improving EVs local retention in bone regeneration

With the understanding of the significant roles of EVs in bone regeneration, EVs have been widely applied to aid in bone repair through various administration routes. The most common mode of EV delivery for tissue repair is via systemic intravenous injection of free EVs or local injection of EVs directly into injury sites [34]. However, EVs exhibit a limited duration of retention, typically lasting for approximately 1 d. A significant portion, ranging from 50 % to 60 %, is promptly cleared from the treatment site within the initial 4-h period post-administration [10]. Thus, there is a critical need to develop reliable and efficient methods to improve EV retention, thereby enhancing their therapeutic potential [35]. In this context, the use of biomaterials for EV delivery has emerged as a promising strategy in regenerative medicine, aiming to achieve the desired concentration of EVs for therapeutic purposes [36]. Recent studies have demonstrated that by binding EVs to biomaterials, their retention and bioavailability following delivery can be extended, enabling sustained and controlled release. This approach significantly enhances the therapeutic potency of EVs (Table 1). Particularly noteworthy are studies by Wang [37] and Mardpour [38], which compared the therapeutic efficacy between local injection of free EVs and utilizing biomaterials for immobilizing and delivering EVs. Their findings unequivocally indicated that biomaterials-based EV delivery offers significant advantages over traditional EV injections, notably in enhancing retention and targeted delivery of EVs to the injury site.

**Table 1:** Summary of EVs-functionalized biomaterials for bone regeneration in vivo.

Source	Biomaterials	Methods	Mechanism	Ref.
hBMSCs	Atelocollagen sponges	Surface adsorption	Increased osteogenesis by aiding in angiogenesis	[115]
rBMSCs	DBM scaffolds	Chemical immobilization	Increased osteogenesis by aiding in angiogenesis	[53]
rBMSCs	Alginate-PCL	Physical encapsulation	Increased osteogenesis by aiding in angiogenesis	[94]
rBMSCs	β-TCP	Surface adsorption	Increased osteogenesis and angiogenesis via carrying mutant HIF-1α	[116]
rBMSCs	Autohydrogel	Physical encapsulation	Increased osteogenesis by inducing biomineralization	[13]
ATDC5	Core-shell nanofibers of CS/PLA	Chemical immobilization	Increased osteogenesis by aiding in angiogenesis through delivering VEGF gene	[117]
hMSCs	PLL-coated 3D Ti-scaffolds	Electrostatic adsorption	Increased osteogenesis by altering miRNAs expression to activate the PI3K/Akt and MAPK signaling pathways	[51]
hMSCs	Ti scaffolds	Surface adsorption	Upregulated the MSC expression of SDF-1α	[14]
hiPS-MSCs	β-TCP	Surface adsorption	Increased osteogenesis by activating PI3K/Akt signaling pathway	[47]
hUCMSCs	Hyaluronan/heparin hydrogels	Physical encapsulation	Increased osteogenesis by aiding in angiogenesis via containing HIF-1α	[44]
hADSCs	pDA-modified SF/PCL	Chemical immobilization	Increased osteogenesis by aiding in angiogenesis	[118]
hADSCs	PLGA/pDA	Chemical immobilization	Increased osteogenesis by promoting MSCs migration and homing	[16]
hADSCs	GNPs hydrogel	Electrostatic interaction	Regulated macrophage phenotypic polarization via containing miR-451a	[30]
hADSCs	Ti implants	Surface adsorption	Promoted osteogenesis-related gene expression	[119]
hADMSCs	Glycosan hydrogel	Physical encapsulation	Increased osteogenesis via containing miR-375	[120]
hGMSCs	3D-printed PLA scaffolds	Electrostatic adsorption	Promoted osteogenesis-related gene expression	[78]
hDPSCs	Chitosan hydrogels	Physical encapsulation	Regulated immune environments containing miR-1246	[32]
hDPSCs	PLGA-PEG-PLGA microspheres	Physical encapsulation	Increased bone formation by recruiting endogenous cells to the defects	[121]
hPLSCs	Collagen membranes	Electrostatic adsorption	Promoted osteogenesis-related gene expression	[77]
hPLSCs	3D collagen membranes	Electrostatic adsorption	Increased osteogenesis by aiding in angiogenesis	[93]
hSHEDs	Bioinspired porous microspheres	Physical encapsulation	Increased osteogenesis by aiding in angiogenesis	[46]
Endothelial cells	Hyaluronic-acid-based hydrogels	Physical encapsulation	Regulated macrophage phenotypic polarization	[122]
Murine osteoclasts	Collagen	Physical encapsulation	Increased osteogenesis by activating TGFβ1 and SMAD3 signaling via containing SPP1	[6]
Murine osteoclasts	DBM scaffolds	Chemical immobilization	Increased osteogenesis and bone mineralization via containing miR-106a-5p	[123]
Murine osteoclasts	DBM scaffolds	Chemical immobilization	Increased osteogenesis and bone mineralization via containing miR-324	[60]
Murine osteoclasts	DBM scaffolds	Chemical immobilization	Increased osteogenesis via RANKL reverse signaling	[58]
Murine osteocytes	PCL scaffold coating with ECM proteins	Chemical immobilization	Not indicated	[63]
Murine osteocytes	Melt electrowritten scaffolds	Chemical immobilization	Not indicated	[124]
Murine macrophages	Collagen scaffolds	Physical encapsulation	M1 MEVs with miR-155 negatively impact BMP2 signaling pathway, while M2 MEVs miR- 378a positively impact it.	[99]
Murine macrophages	Hyaluronic acid-alginate hydrogels	Physical encapsulation	Improved the immunomodulatory, osteogenic and angiogenic ability	[45]
Macrophages	Ti nanotubes	Surface adsorption	Increased osteogenesis via activation of autophagic activity	[49]

## 4 Methods to integrate EVs into bioactive scaffolds

**Fig. 2:**
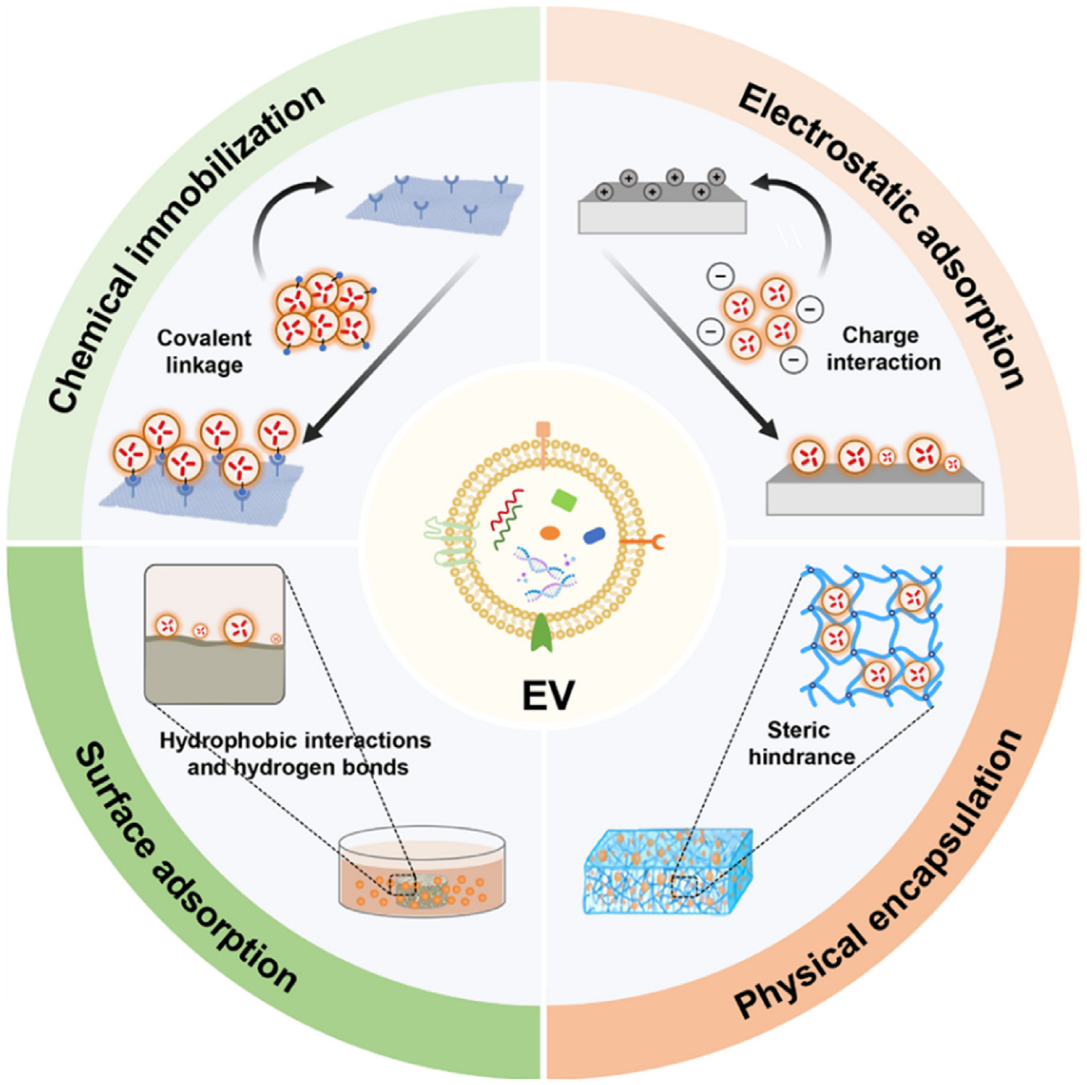
Different methods for incorporating EVs into biomaterials. The diagram illustrates four methods for incorporating EVs into biomaterials: physical encapsulation, surface adsorption, electrostatic interaction, and chemical immobilization. In physical encapsulation, EVs are simply entrapped within three-dimensional scaffolds, and the diffusion of EVs is influenced by the steric hindrance of the scaffolds. For surface adsorption, scaffolds are immersed in a solution containing EVs, facilitating the attachment of EVs to the surfaces of biomaterials through hydrophobic interactions and hydrogen bonds. In the case of electrostatic interaction, electronegative EVs bond to bioactive scaffolds with positive charges. Chemical immobilization involves covalently grafting active groups onto EV surfaces and conjugating targeting ligands, such as antibodies and peptides, to the surfaces of biomaterials. This enables the specific immobilization of EVs onto the scaffolds.

By leveraging different physiochemical characteristics of EVs, such as negative charge, lipid, and protein content, various methods have been developed accordingly for the incorporation of EVs into biomaterials. These methods include physical encapsulation, surface adsorption, electrostatic interaction, and chemical immobilization to scaffolds through ECM proteins, adhesion molecules, and targeted proteins or ligands (Fig. 2).

### 4.1 Physical encapsulation

The physical constraints imposed by 3D scaffolds necessitate straightforward entrapment of EVs within polymers and hydrogels, such as alginate, chitosan, pluronic, and silk-based hydrogels. By changing the composition, porosity, degradation rate, and swelling rate of these materials, the release kinetics of EVs can be regulated [39,40]. For example, adjusting the pore size of hydrogels allows for differential release rates of EVs [41,42]. Moreover, by changing the concentration or cross-linking modes of alginate hydrogels, the diffusion of EVs was significantly affected due to differences in the steric hindrance of the alginate polymer chains [13,[43], [44], [45]]. In particular, injectable hydrogels have gained significant attention as an effective way of precisely delivering EVs to desired sites and sustaining the release of encapsulated EVs [12]. For instance, when human placenta MSC-derived EVs were incorporated in thermosensitive chitosan hydrogels, real-time monitoring revealed considerably improved retention and stability and sustained release of EVs over a period of 30 d in vivo [35]. In addition to bulk hydrogels, nanogels and microspheres have also been employed for the sustained release of EVs because they typically have a more uniform size and shape and higher surface area-to-volume ratio, which can load more EVs and own more consistent release profiles. For instance, microspheres made of poly(lactide-co-glycolic acid) (PLGA) with an interconnected porous structure were employed to encapsulate EVs and demonstrated effective accumulation at the injury sites over a 21-d period [46].

### 4.2 Surface adsorption

The other most straightforward method to incorporate EVs into scaffolds is through surface adsorption. This method involves incubating scaffolds in a solution of EVs, allowing them to adhere to the scaffold surface through hydrogen bonding or hydrophobic interactions. Unlike other methods, surface adsorption is simple and does not require chemical grafting reagents or alterations to the EV structure (Table 2). However, one limitation of this method is the potential for burst release, where a large portion of the loaded EVs may be rapidly released from the scaffold surface. Despite this drawback, significant improvements in bone formation were observed in various studies via surface adsorption. For example, Zhang et al. [47] integrated human-induced pluripotent stem cell (hiPS)-MSCs-derived EVs with β-tricalcium phosphate (β-TCP) particles simply by immersing these particles in an EV solution for 4 h. Despite having burst release, this cell-free system still significantly stimulated bone formation in a rat calvarial defect model [47]. Similar improvements in bone repair by EVs via simple surface adsorption were found using PLGA scaffolds loaded with EVs-derived BMSCs [48] and titanium (Ti) scaffolds loaded with macrophage-derived and platelet lysate-derived EVs [49,50].

**Table 2:** Summary of different methods to integrate EVs with bioactive scaffolds.

Method	Advantages	Disadvantages
Physical encapsulation	Easily manipulated, high loading capacity	Low controlled release ability
Surface adsorption	Simple and straightforward preparation	Low controlled release ability
Electrostatic interaction	High stability and controlled release ability	Susceptible to destabilization
Chemical immobilization	Sustained release kinetics	Risk of EV structure and function damage

### 4.3 Electrostatic interaction

Electrostatic interaction between EVs and the surface of biomaterials is an important factor in the delivery of EVs. Due to the negatively charged phospholipid membranes, EVs have a zeta potential of approximately −30.8 mV [15]. This negative charge allows EVs to be immobilized within biomaterials through electrostatic interaction. EV-functionalized scaffolds, through electrostatic interactions, often exhibit enhanced stability compared to those functionalized via physical encapsulation and surface adsorption, protecting the EVs from degradation or denaturation. More importantly, by modifying the charge of scaffolds, electrostatic interactions can facilitate controlled release of EVs to improve their therapeutic efficacy. However, it is of note that electrostatic complexes may be susceptible to destabilization in altered pH or the presence of competing ions, which can affect the efficacy of EV delivery. Zhai et al. [51] employed a method to coat a 3D Ti scaffold with poly-l-lysine (PLL), a biocompatible polymer with a positive charge. Following the coating process, MSC-derived EVs were introduced to the PLL-coated Ti scaffolds. The electrostatic interaction between PLL and EVs facilitated the adsorption of EVs onto the PLL-coated Ti scaffolds. Notably, this method achieved an impressive EV loading efficiency of 79.48 % after 12 h of incubation in the PLL solution. [51]. In another study, polycaprolactone (PCL) fibers were chemically modified with positively charged polyethylenimine (PEI) [15]. This modification facilitated electrostatic interaction with negatively charged exosomes, enabling their tethering onto the PCL fiber surfaces and realize a controlled release of 50 % EVs for more than 7 d.

### 4.4 Chemical immobilization

Compared to regular physical methods, covalent linking provides a more efficient way to functionalize scaffolds with EVs. Proteomic studies have identified a wide range of proteins within the exosomal membrane, including cytoskeletal components (actin, cofilin, tubulin), membrane fusion proteins (annexins, Ras-associated binding proteins), as well as other membrane-associated proteins such as integrins, tetraspanins, and lactadherin. By conjugating targeting ligands such as antibodies and peptides to the surface of biomaterials, the specific immobilization of EVs with scaffolds can be achieved. Integrins, for example, play a role in interacting with the surrounding ECM environment [34,52]. Therefore, coating biomaterials with ECM proteins, such as fibronectin and type I collagen, can provide an adhesive surface for integrating EVs onto certain scaffolds [53]. EVs can also be conjugated to the surface of scaffolds by leveraging the specific interaction between surface molecules of EVs and adhesion molecules or targeted proteins. For example, polydopamine (pDA), known for its adhesive properties, can bind to various substrates for biomedical applications. By adhering to pDA, EVs can be carried and retained, enabling a slow and localized release [16]. Additionally, targeted proteins such as the anti-CD63 antibody and integrin α4β1 ligand (LLP2A) can specifically bind to EVs [34]. The anti-CD63 antibody-coated electrospun nanofibrous scaffolds and LLP2A immobilized polymer scaffolds, for instance, can specifically bind to hASC-derived EVs [17,54]. However, we must keep in mind that covalent linkage strategies often involve chemical modifications of EV surface proteins or lipids to attach cargo molecules. These modifications can potentially disrupt the natural structure and function of EVs, leading to altered behavior and reduced efficacy in intercellular communication. Besides, chemical modifications may introduce foreign molecules or residues onto the EV surface, increasing the risk of immune recognition and undesirable side effects.

## 5 Various types of EVs used in bone regeneration

**Fig. 3:**
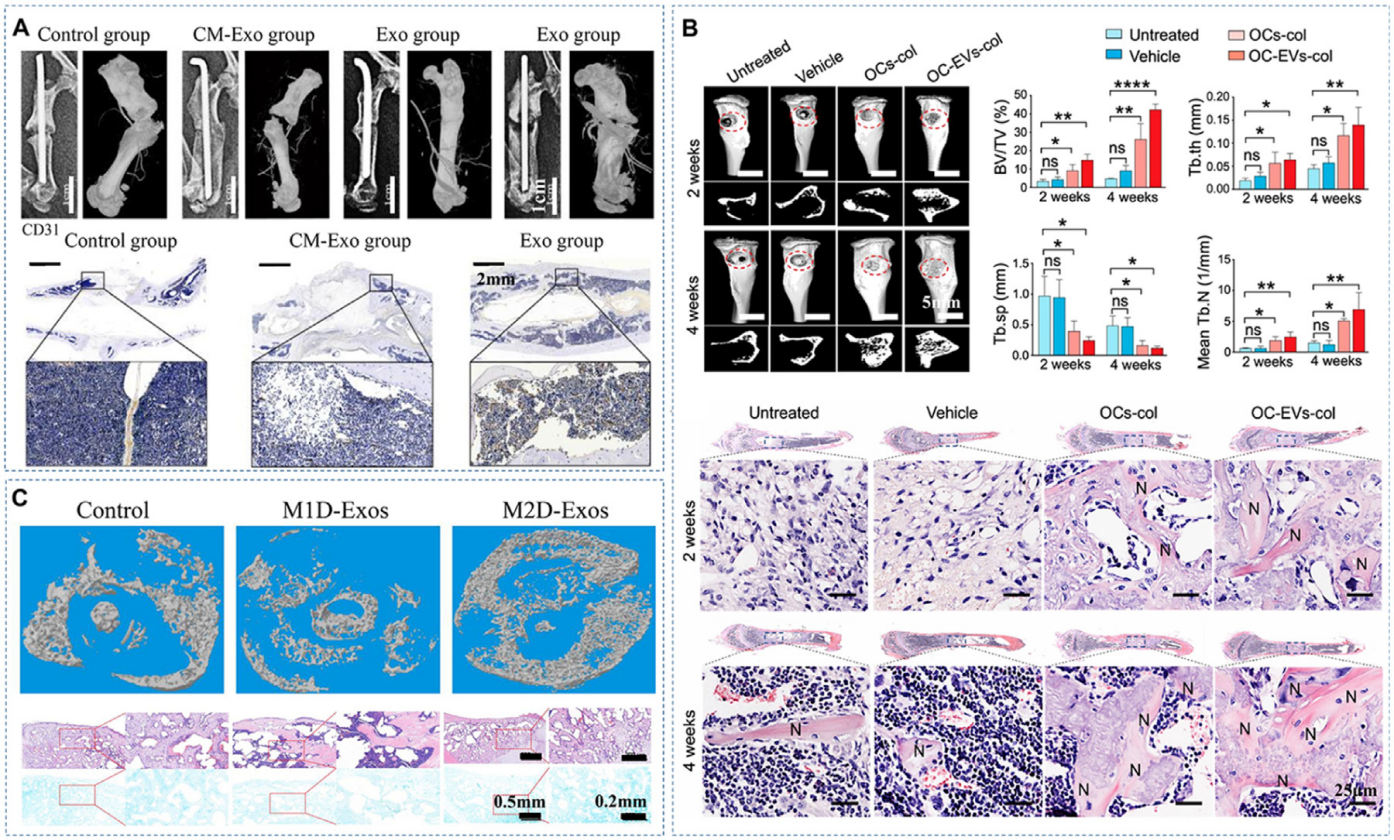
EVs from different sources incorporated into bioactive scaffolds for bone repair. (A) EVs derived from BMSCs significantly enhanced femur bone fracture repair compared to exosome-depleted groups after 20 weeks. Adapted with permission. Copyright 2020, BioMed Central [113]. (B) Osteoclast EVs demonstrated an augmentation of tibia bone defect regeneration compared to the vehicle group after 4 weeks. Adapted with permission. Copyright 2023, Elsevier Inc [6]. (C) EVs from M2 macrophages exhibited notable potential in accelerating murine femur fracture healing compared to EVs from M1 macrophages after 3 weeks of treatment in bone fracture scenarios. Adapted with permission. Copyright 2021, BioMed Central [28].

Bone remodeling is a complex and continuous process that relies on well-regulated communication among various bone-related cells through EVs [19]. Therefore, EVs from these osteogenic, angiogenic, and immune cells are frequently used to augment bioactivities of scaffolds to enhance bone formation (Fig. 3). Beyond traditional sources like mammalian cells, researchers are exploring EVs derived from various plant cells, biological fluids, and microorganisms, as novel sources of EVs for bolstering bone repair.

### 5.1 EVs from MSCs

Due to the extensive investigation of MSC-based products in tissue engineering, EVs from different tissue-derived MSCs, including bone marrow, adipose tissue, and oral tissue like dental pulp, gingival, and periodontal, have been explored for bone regeneration. Fan et al. [55] demonstrated that EVs from BMSCs can be phagocytosed by macrophages and promote M2 macrophage polarization via the NF-κβ pathway. This further enhances osteogenic differentiation of BMSCs in vitro and bone formation. Human adipose derived mesenchymal stem cells (hASCs) have also shown promise as a source of EVs because of their low donor-site morbidity and high yield efficiency. Liu et al. [56] incorporated hASC-derived EVs into PLGA scaffolds and found increased bone regeneration capacity via its osteoinductive effects in mouse calvarial defects. Moreover, iPSCs have attracted much attention recently because they are non-invasively harvested and can be autologously transplanted without immune rejection. A study by Zhang et al. [47] showed that iPSC-derived EVs notably enhanced the osteogenic differentiation of BMSCs in vitro. Furthermore, the study substantiated the pro-osteogenic effects of iPSC-derived EVs in vivo by loading them into β-TCP granules in a rat critical-sized calvarial defect model.

### 5.2 EVs from osteoclasts

The crosstalk between bone-resorbing osteoclasts and bone-forming osteoblasts, known as ‘‘coupling’’, plays crucial roles in bone remodeling. Osteoclast-derived EVs have emerged as novel regulators in modulating this coupling [23]. For example, apoptotic bodies released from osteoclasts have been shown to be engulfed by preosteoblastic cells, promoting osteoblast differentiation in vitro [57]. Incorporating osteoclasts-derived EVs into a DBM scaffold induced osteogenic differentiation of MSCs and increased bone formation in mouse cranial defects via activation of RANKL reverse signaling [58]. This team also found that preosteoclasts-derived apoptotic bodies can induce the vascularization in the bone regeneration process by inducing endothelial cell differentiation and vessel formation via their Platelet-Derived Growth Factor-BB (PDGF-BB) cargoes [59]. Additionally, osteoclast-derived small EVs have also been found to induce osteogenic differentiation and mineralization of MSCs by inhibiting ARHGAP1, a negative regulator of osteogenic differentiation [60]. Incorporating these EVs into scaffolds significantly promotes bone regeneration.

### 5.3 EVs from osteocytes

Osteocytes, as one of mechanosensitive cells, play multifunctional roles in the regulation of bone remodeling [61]. When myostatin-modified osteocytic EVs were co-cultured with osteoblasts, they were taken up by osteoblastic cells and inhibited osteoblastic differentiation by down-regulating the Wnt signaling pathways [62]. Osteocytes can also secrete cytokines such as sclerostin and osteoprotegerin (OPG), as well as EVs, to regulate bone metabolism responding to mechanical stimuli [25]. Likewise, Nieuwoudt [63] demonstrated that scaffolds containing osteocyte-derived EVs under mechanical activation significantly enhance MSC osteogenesis and repair damaged bone.

### 5.4 EVs from immune cells

Intercellular communication between immune cells and bone cells is an active element in the bone remodeling and regeneration [19]. EVs released from immune cells, such as macrophages [28], monocytes [64], and dendritic cells [27], have proved to be of vital importance in bone regeneration. For example, Liu et al. [65] demonstrated that EVs from biomimetic collagen-treated macrophages facilitated osteogenesis through the canonical BMP2/Smad5 pathway. Similarly, EVs from BMP2-stimulated macrophages can be endocytosed by BMSCs, and incorporating BMP2-stimulated macrophages-derived EVs into Ti nanotubes notably promoted the osteoblastic differentiation of MSCs [49]. Except for macrophages, Ekstrom et al. [64] demonstrated that lipopolysaccharide-stimulated human monocyte-derived EVs were internalized by MSCs and enhanced the osteogenic differentiation of BMSCs with an increase of the osteocalcin expression. Dendritic cell-derived EVs, on the other hand, may not directly influence the osteogenic differentiation of MSCs but have been shown to promote the recruitment and migration of endogenous cells, thereby facilitating bone regeneration [66].

### 5.5 EVs from emerging sources

In addition to EVs derived from mammalian cells, novel sources of EVs from various plant cells, bodily fluids, such as milk, blood and urine, as well as those produced by bacteria, have emerged as promising candidates for enhancing bone repair. Among these, plant-derived EVs (PDVs) have garnered attention. These PDVs typically range in diameter from 50 to 1000 nm and are enriched with distinctive bioactive compounds. These include enzymes involved in cell wall remodeling, proteins exhibiting antimicrobial properties, and small non-coding RNAs [67]. PDVs demonstrate potential antioxidant, anti-inflammatory, and anti-resorptive effects, thereby promoting bone formation [68]. Integrating PDVs into scaffold materials or employing them as independent therapeutic agents shows considerable promise in enhancing the efficacy of bone repair and regeneration endeavors [[69], [70], [71]]. Additionally, bacterial EVs from probiotics like *Akkermansia muciniphila* are found to contain a rich array of nucleic acids, proteins, lipopolysaccharides and peptidoglycan, which have shown potential in enhancing bone mass and strength in ovariectomy-induced osteoporotic mice by augmenting osteogenic differentiation and inhibiting osteoclast activity [72].

EVs are abundant in various biological fluids, including milk, blood, urine, saliva etc. [73]. EVs present in these fluids also affect bone homeostasis. Milk-derived EVs were reported to facilitate bone repair by promoting the expression of the osteogenic gene GJA1 [74]. Meanwhile, blood and urine-derived EVs have garnered significant attention as they can serve as diagnostic markers for bone diseases. More recent studies found that blood and urine-derived EVs are enriched in different microRNAs, which can ameliorate bone loss in senile osteoporotic mice [75,76].

## 6 Biomaterials used to deliver EVs locally

**Fig. 4:**
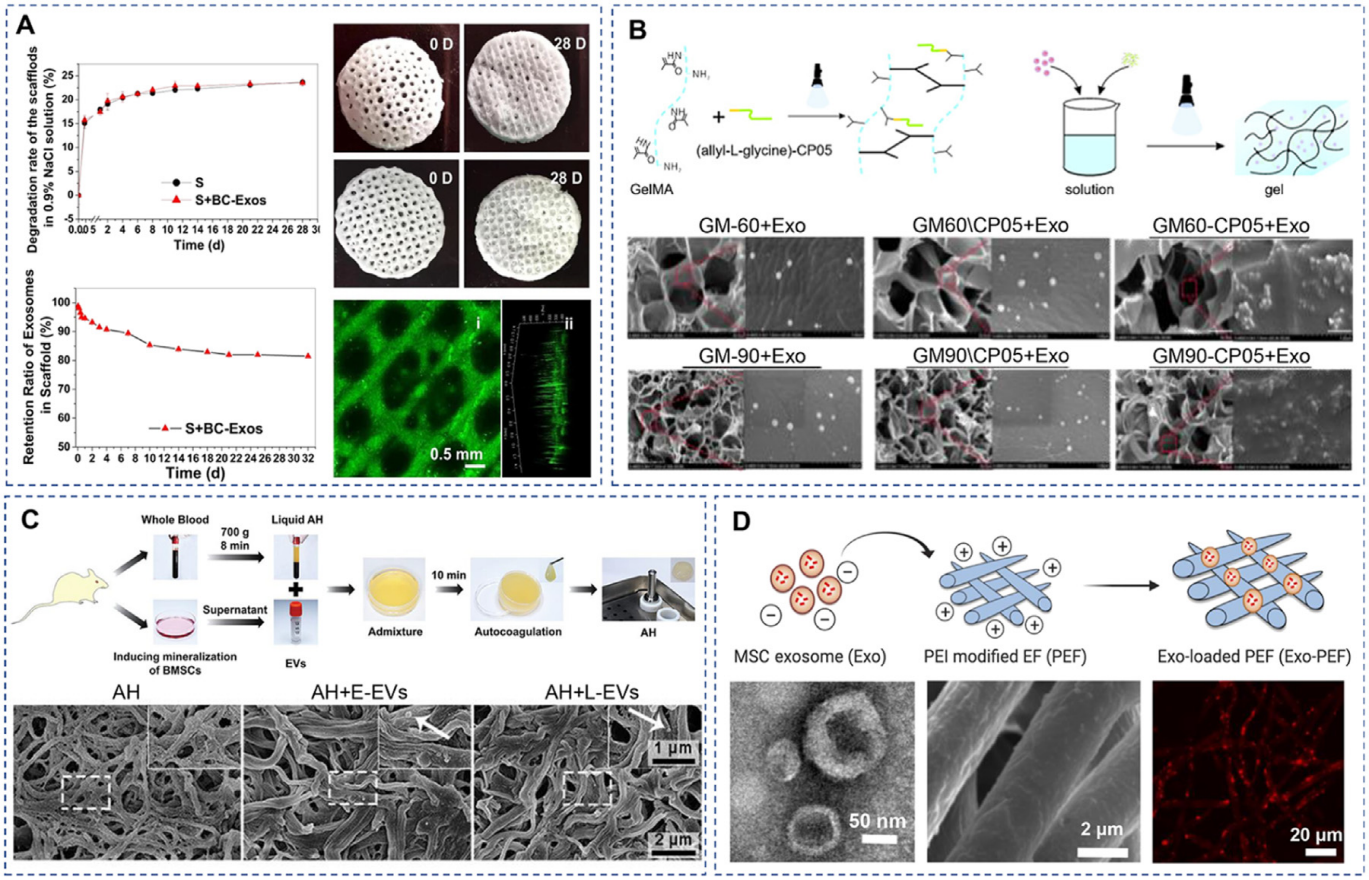
Biomaterials used for EV delivery for bone repair. (A) 3D-printed β-TCP scaffold loaded with EVs through surface adsorption ensured sustained release of EVs over a period of one month. Adapted with permission. Copyright 2021, Springer Science and Business Media LLC [45]. (B) GelMA hydrogels and (C) autohydrogels incorporated EVs via physical encapsulation and achieved a complete cumulative release in a short time. Adapted with permission. Copyright 2023, BioMed Central [114]. Adapted with permission. Copyright 2020, Wiley-VCH [13]. (D) Polyethylenimine-modified electrospun fibers, functionalized with EVs through electrostatic interactions, exhibited prolonged release kinetics over one week. Adapted with permission. Copyright 2021, AAAS [15].

Biomaterials used for EV delivery in bone repair can be categorized into three main types: natural biomaterials, synthetic biomaterials, and chemically modified or hybrid biomaterials (Fig. 4). Natural biomaterials, such as collagen, hyaluronic acid, and alginate can provide cell-binding sites that support bone ingrowth. Moreover, encapsulating EVs in natural hydrogels can provide protection against rapid clearance and enhance membrane integrity and mechanical stability. For example, EV binding to collagen membranes via integrins showed high biocompatibility and osteogenic potential in both cell culture and animal studies [77]. Synthetic biomaterials such as Ti and Ti-based alloys and bioceramics like β-TCP are widely used as scaffolds for bone regeneration owing to their excellent mechanical properties. Due to their bio-inert properties, EVs are usually loaded onto these biomaterials through surface adsorption. However, conventional physical adsorption techniques do not achieve high loading efficiency or allow for controlled release of EVs. Synthetic polymer biomaterials, such as poly(lactic acid) (PLA), PCL, and PLGA, have also been used to deliver EVs for bone regeneration due to their common characteristics, such as biodegradability, biocompatibility, and specific structures. For example, Diomede et al. [78] showed that PLA scaffolds functionalized with human gingival stem cell-derived EVs provided a favorable endogenous microenvironment to support cell survival, proliferation, and differentiation. Moreover, natural biomaterials can be chemically modified to introduce desirable biological properties and tunable physical characteristics. Gelatin- methacryloyl (GelMA) is an example of a chemically modified natural biomaterial that has been explored for EV delivery in bone regeneration. For instance, MSC-derived EVs incorporated into GelMA enabled sustained release of EVs for over 14 d and significantly enhanced the ECM collagen production and mineralization of BMSCs [79]. Nonetheless, these synthetic materials still need to be modified to meet the physiochemical properties of bioactive scaffolds for bone regeneration.

To address these limitations, hybrid bioactive materials composed of natural and synthetic materials have been investigated to load EVs for bone regeneration [16]. For example, Yang et al. [80] prepared hydroxyapatite-hyaluronic acid-alginate hydrogels with robust mechanical properties and desirable biocompatibility as a vehicle for EV delivery. These composite hydrogels effectively retain EVs at the defect sites for more than two weeks and significantly increased new bone formation compared to hydrogels without EVs.

## 7 Mechanisms of EV-functionalized bioactive scaffolds for bone formation

**Fig. 5:**
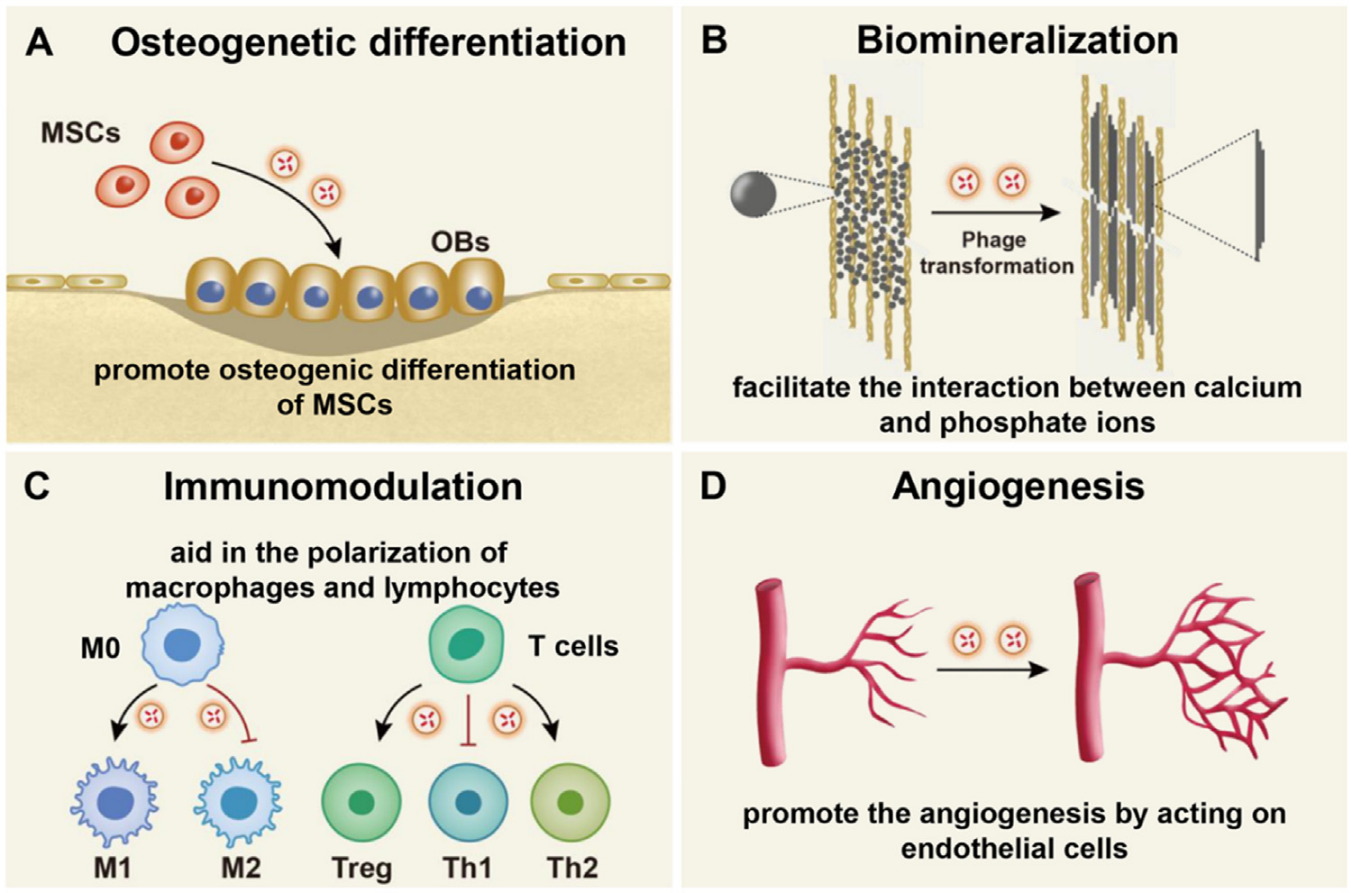
Schematics of the mechanisms of EVs promoting bone regeneration. (A) EVs promote the osteogenic differentiation of MSCs through miR-27a, miR-21, miR-217 and miR-26a etc. (B) EVs promote bone regeneration by aiding in the biomineralization process through facilitating the interaction between calcium and phosphate ions through annexin V, alkaline phosphatase, and nucleotide pyrophosphatase phosphodiesterase. (C) EVs regulate the immune response by polarizing macrophages and lymphocytes through miR-451a, miR-1246 and miR-378a. (D) EVs contributed to bone regeneration by increasing angiogenesis by acting on endothelial cells through VEGF, bFGF and miR-27b.

Further investigations into the underlying mechanisms by which EVs promote bone regeneration can serve as inspiration for developing novel EV-functionalized biomaterials and improving their therapeutic efficacy. Consequently, we summarize the reported mechanisms through which EV-functionalized scaffolds facilitate bone regeneration (Fig. 5).

### 7.1 Promoting osteogenic differentiation

Osteogenic differentiation is a critical process in bone generation, and research has shown that EVs from various cell sources can induce osteogenic differentiation to repair bone defects. Significant research has focused on the effects of EVs from MSCs in stimulating osteogenic differentiation. Upregulated miRNA such as miR-21, miR-26a, miR-27a, miR-217 [56,[81], [82], [83]] and downregulated expression of miR-23a, miR-23b, and miR-124 [[84], [85], [86], [87]] in EVs derived from MSCs have been identified as key factors in promoting osteogenic differentiation. Additionally, osteoclasts, conventionally associated with bone resorption, have also been found to play a role in osteogenic differentiation. For instance, Liang et al. [60] demonstrated that osteoclast-derived EVs induced osteoblastic differentiation of MSCs by targeting a negative regulator of osteogenic differentiation via miR-324. Incorporating miR-324-enriched EVs into a scaffold significantly enhanced bone regeneration in a mouse calvarial defect model [60].

### 7.2 Promoting biomineralization process

EVs contain calcium phosphate droplets, membrane proteins, and enzymes, such as annexin V, alkaline phosphatase, and nucleotide pyrophosphatase phosphodiesterase, which can promote bone regeneration by directly facilitating matrix mineralization [88]. In particular, matrix vesicle proteins and lipids serve as nucleation sites for crystal deposition [89]. Besides, matrix enzymes regulate the ratio of inorganic phosphate to inorganic pyrophosphate to control the matrix mineralization. Davies et al. [90] observed that MSC-derived EVs had augmentation in mineralization content, elevated levels of annexin proteins involved in phospholipid binding and calcium channeling, as well as modifications in nucleation capacity. On this basis, Wei et al. [13] demonstrated that EVs of MSCs promoted the nucleation of extracellular mineral crystals, leading to in situ self-mineralization to aid in bone regeneration. Through similar mode of action, EVs from osteoblastic cells were functionalized with cell-specific aptamers and then leveraged to stimulate bone regeneration with black phosphorus [91].

### 7.3 Regulating immune response

EVs have been shown to play a role in modulating immune responses and promoting bone regeneration [15,32,55]. For instance, EVs derived from ASCs loaded into gelatin nanoparticle-based hydrogels can polarize macrophages towards the M2 phenotype. This is accomplished by repressing macrophage migration inhibitory factors via miR-451a carried by ASCs. This immunomodulatory effect from ASCs-derived EVs significantly enhanced new bone formation after incorporation into gelatin hydrogels [30]. Furthermore, Su et al. [15] found that polyester materials incorporating MSC-derived EVs can act as the trainer for immune cells. These EV-containing polyester materials regulated the immune response by promoting the desired macrophage phenotype and regulatory T cells. Another interesting study demonstrated that dental pulp stem cell (DPSCs)-derived EVs, when incorporated into chitosan hydrogels, could mitigate periodontal inflammation and expedite the healing of alveolar bone [32]. In this process, miR-1246 from the EVs primarily contributes to the immunomodulatory effects on macrophages and further bone formation.

### 7.4 Enhancing angiogenesis

Angiogenesis is a key process in bone repair since the newly formed blood vessels supply nutrients, oxygen, minerals, growth factors, and progenitor cells for bone formation [92]. Recent studies have demonstrated the role of EVs in promoting angiogenesis and bone regeneration [44,93,94]. Zhang et al. [44] highlighted that EVs released from umbilical cord mesenchymal stem cells (UCMSCs) possess the ability to enhance the angiogenic potentail of endothelial cells and promote angiogenesis in vivo. EVs derived from human periodontal-ligament stem cells integrated into collagen membranes also showed to increase vascularization in bone defects by upregulating the expression of vascular endothelial growth factor (VEGF) and VEGFR2 to facilitate angiogenesis and osteogenesis [93]. Utilizing this proangiogenic potential, Xie et al. [94] loaded MSC-derived EVs into alginate-PCL scaffolds and obtained significantly increased blood vessels and bone regeneration in murine subcutaneous tissues. EV components such as Interleukin 6 (IL-6), basic fibroblast growth factor (bFGF), angiogenin, and monocyte chemotactic protein 1 (MCP-1) have been implicated in promoting angiogenesis. Except for EV-derived proteins, specific miRNAs in EVs such as miR-125a, miR-126, and miR-214, were also shown to play important roles in mediating EV-induced angiogenesis as part of the bone regeneration process [95].

## 8 Limitations and future perspectives

**Fig. 6:**
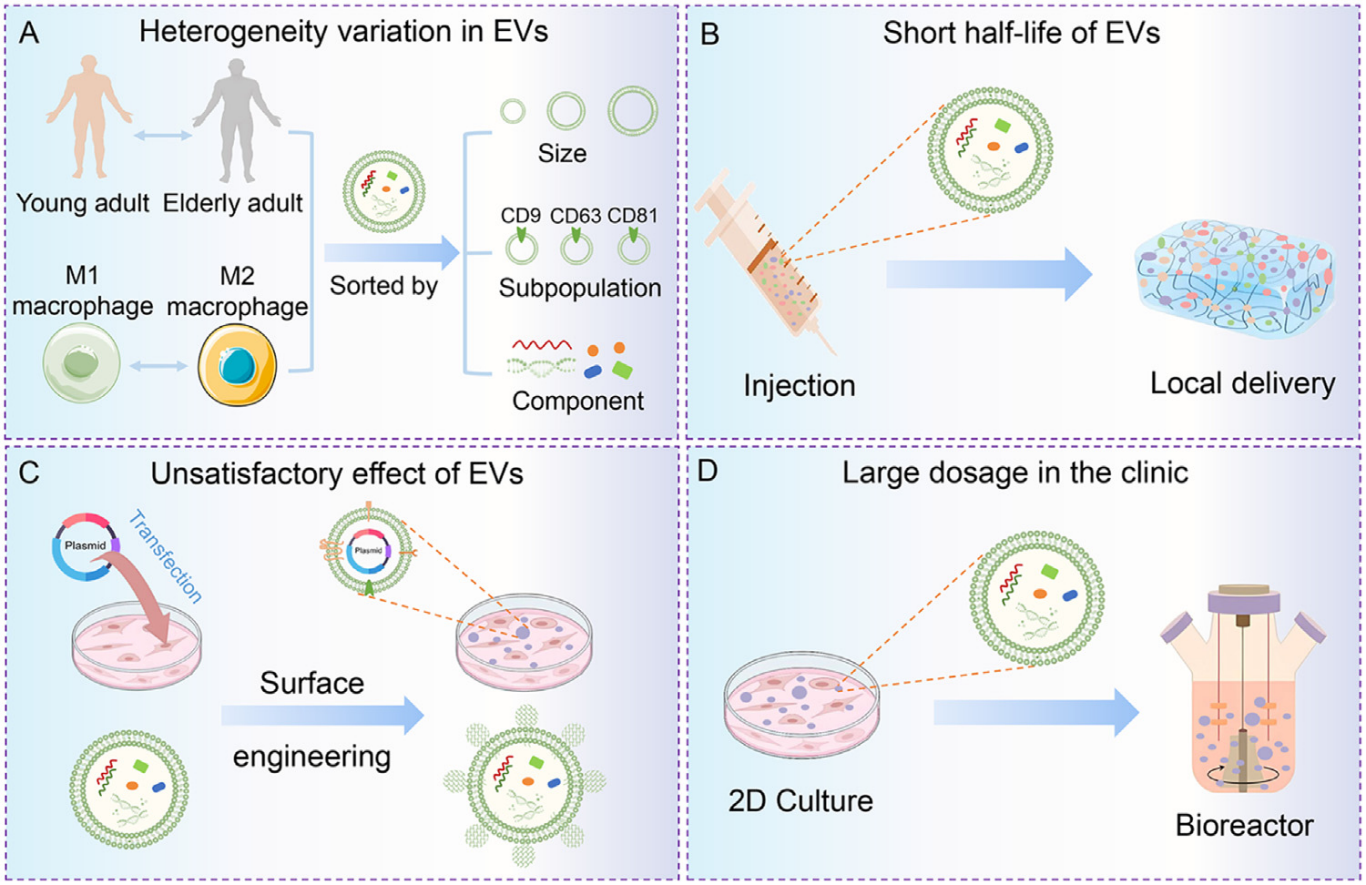
Summary of current challenges and potential solutions for the clinical application of EVs. (A) EVs from various sources have heterogeneity. This can be addressed by sorting EVs based on specific characteristics such as size, protein markers, and other components of EVs. (B) EVs after direct injection have a short-life span. Local delivery using biomaterials can increase the retention and concentration of EVs. **(C)** Current therapeutic applications of EVs are restrained by their unsatisfactory effects in compromised conditions. Surface engineering and gene modification strategies can be employed to enhance the effectiveness of EVs. (D) Clinical applications of EVs often require large doses. Utilizing bioreactors and well-designed vehicles can be considered to increase the production of EVs.

Despite considerable achievements in the use of EV-functionalized bioactive scaffolds for bone repair and regeneration, the potential limitations should also be considered before clinical application of EVs (Fig. 6). From a practical view, the composition of EVs varies significantly depending on factors such as the age, type, and the state of parent cells, as well as the surrounding physiological, physical, chemical, and mechanical environments [96]. Therefore, a full characterization of EVs under different conditions is crucial for understanding their underlying mechanisms, which in turn influence their therapeutic efficacy in bone regeneration. For example, EVs from old patients compared to young patients or from M1 macrophages compared to M2 macrophages have been found to have disparate effects on the osteogenic differentiation of MSCs and bone repair capacity [[97], [98], [99]]. Therefore, it requires comprehensive assessment of EV functions, pharmacokinetics, and the possible mechanisms of action of EVs for future therapeutic development [100].

Bone regeneration generally requires a relatively long healing time. However, maintaining the presence of EVs and their therapeutic effects at injury sites over such a prolonged period can be challenging. To enhance the therapeutic efficacy of EV-modified scaffolds, it is important to explore appropriate EV delivery systems. Therefore, future research should prioritize the further refinement of EV-functionalized bioactive scaffolds, including considerations of degradation profiles, physical architecture (pore size, porosity, etc.), loading density, and release profiles. For example, sustained delivery of EVs can be realized by incorporating EVs into microgels or microspheres [101,102]. These carriers enable the homogeneous distribution of EVs for controlled release. Moreover, the release profile can be easily altered by adjusting characteristics of the microgels, such as composition, size, morphology, and dispersion. Another clarified aspect is the mechanism by which EVs are released from these EV-functionalized scaffolds in vivo. To address this, advanced molecular imaging techniques should be employed to enable highly specific and high-resolution tracking of EVs in real-time.

Despite extensive evidence supporting the beneficial effects of EV-functionalized scaffolds on bone repair, their therapeutic efficacy in large-size bone defects and pathological conditions remains to be fully determined. These conditions generally require an enhanced therapeutic potential of EVs than that under normal conditions. Several techniques have been attempted to improve the therapeutic efficacy of EVs. One direct approach involves genetically modifying the EV-producing parent cells to engineer EVs. Another approach is the binding of targeting proteins or aptamers to the surfaces of EVs [103]. For instance, PEI, a positively charged biocompatible polymer, has been leveraged to immobilize negatively charged EVs [104]. The other strategy is to use engineered EVs to more specifically target bone surfaces or bone cells, such as osteoblasts and osteoclasts. Moreover, the internal cargoes of EVs can be manipulated by changing the cell culture microenvironment. For instance, oxygen levels or nitric oxide stimulation can significantly alter the components, quantities, and therapeutic potential of EVs [105,106]. Besides, the stimulation of MSCs using tumor necrosis factor-α (TNF-α) or Dimethyloxaloylglycine also significantly improved the osteogenic potential of MSCs-derived EVs [31,107]. More notably, bone cells such as osteoblasts, osteocytes, and osteoclasts can response to mechanical stimuli and change their behavior, including the components and therapeutic efficacy of their EVs [108]. Morell et al. [109] presumed that mechanical stimulation of osteocytes activated the release of EVs containing bone regulatory proteins and thereafter initiated the bone formation process. Therefore, the utilization of EVs from cells under appropriate mechanical stimuli has great potential for aiding bone regeneration, given the simplicity and low cost of this approach.

To date, clinical trials investigating the use of EVs to enhance bone repair remain limited. One of the key challenges associated with EV-based therapeutics is the generally low production yield of EVs [19]. Typically, EV yields are less than 1e+9 particles/ml of culture medium. While tissue culture bioreactors offer an alternative for achieving higher production compared to conventional 2D culture conditions [110], they often fall short of producing a therapeutically useful dose of EVs, which is estimated to be approximately 4e+11 particles per defect. Moreover, there is an ongoing debate on the therapeutic efficacy of EVs produced on a large-scale. For instance, Xie et al. [111] pointed out that EV dosages at 2e+8 particles/ml and 8e+8 particles/ml exhibited lower therapeutic efficacy compared to 4e+8 particles/ml. In another study by Hagey et al. [112] suggests that EVs produced on a large scale may only differ in lysosomal-related functions, lacking significant specificities regardless of their cellular origin. In contrast, differences in EV components from various cell sources are apparent at lower doses, indicating that both the function and quantity of EVs are equally crucial for their therapeutic effects. Furthermore, measuring particle and protein concentrations may not fully reflect EV activity, complicating comparisons between results from different studies. Therefore, a systematic understanding of how to maintain the main functions of EVs during the manufacturing process is necessary. This involves developing identity and potency assays to assess the reproducibility of EV manufacturing and the consistency of EV quality.

## 9 Conclusion

To enhance the therapeutic potential of EVs for bone repair, various techniques, including physical encapsulation, surface adsorption, electrostatic adsorption, and chemical immobilization, can be utilized to integrate them into biomaterials, thus preparing EV-functionalized bioactive scaffolds. Compelling evidence supports the beneficial effects of EV-functionalized scaffolds in bone repair. However, addressing challenges related to EVs’ controlled delivery, large-scale production, and assessing reproducibility in manufacturing is crucial before EV-functionalized bioactive scaffolds can be translated into clinical applications for bone repair.

## Conflicts of interest

The authors declare no conflict of interest.
